# Notes

**Published:** 1900-01

**Authors:** 


### NOTES.

---

HOG INSPECTION IN GERMANY. Dr. Vosburg Rekow and Edmund Wirth, the former a Director of the Imperial Central Bureau of Commercial Treaties of Germany, and the latter a large manufacturer and President of the Chamber of Commerce in Seraw, Brandenburg, have just concluded an inspection of the Chicago packing houses.

They expressed their pleasure and astonishment at the cleanliness of the methods. They admitted that hog-inspection in this country is a great deal better than the German method, because, they say, if any trichniæ are found in Germany the meat after being cooked for a certain period is put on the market ; whereas here if the smallest defect is found the pork is used for by-products.—*Sanitarian*, January, 1900.

The Board of Health of Columbia, S. C., is conducting a prosecution of one of its principal butchers for the slaughter and exposure for sale of a number of carcasses of hogs from a carload shipped into that city, many of which had and subsequently died of hog-cholera. There is entirely too much latitude given certain dealers and butchers in allowing them to go out and openly buy diseased stock or animals from infected premises, and rapidly prepare the same for market. A few prosecutions and vigorous punishment to offenders would have a salutary effect in breaking up this unscrupulous and dangerous practice.

South Carolina has suffered much the past year in great losses of swine from hog-cholera which prevails in many districts. In certain sections of the State where the stock law is rigidly enforced the reduction in outbreaks of Texas fever have been most marked. The cattle tick has disappeared from these sections. Along the coast and southern districts of the State the disease prevails, and cause many losses. The State has no laws for the control of animal diseases, and many loads of cattle from infected districts are shipped to Columbia, Charleston, Richmond, and Norfolk. The last Legislature authorized the State Board of Health to establish such quarantine measures as will protect the live-stock interests of the State, and co-operate with the Department of Agriculture. The State Board of Health has no veterinarian upon its board, and the lack of knowledge of those upon the board makes the law practically a dead letter.

Berlin, Germany, has become quite a centre of distribution of the American heavy draught horses, some of them being shipped from this centre to Italy.

Brood mares are again bringing excellent prices, and reports from all over the country indicate a return to breeding again on a broader and better scale.

Secretary of Agriculture Wilson visited the National Farm School at Doylestown, Pa., during the Christmas holidays. Among those who assisted in entertaining him were State Veterinarian Pearson and Dr. W. G. Benner, who teaches veterinary science at the Farm School.

The British Government has purchased two shiploads of common horses in the North Pacific country for use in South Africa. The horses which went begging for $10 a head last year were sold for $47 each.

The two Arabian stallions which were presented by the Sultan to General Grant when the latter was in Constantinople on his way around the world, are still alive. One of them, Linden Tree, who is now about twenty-five years old, is owned by General Colby, of Beatrice, Neb.

A series of experiments which have been carried on for some time past by the Agricultural Department at Washington have recently developed a new use for cornstalks, from which a very valuable horse and cattle food can now be made by grinding the dry cornstalks, leaves, and tops to a powder and mixing it with blood or molasses or both. This is pressed into cakes under a hydraulic press.

C. W. Williams names his horses alphabetically, and by remembering the year he started it is an easy matter to get at the age of any horse he ever bred. Mr. Williams began breeding in 1885. His first foals came in 1886, and their names all began with "A," as Axtell and Allerton. The next year Barnhart came. The name of every foal he raised ten years later begins with "J," the tenth letter of the alphabet. He has now been in the business fourteen years, and the name of every foal of 1899 begins with "N."

The representatives of the British Government who have been buying mules in Missouri, Texas, and other Southern States and shipping them to South Africa for use in the campaign against the Boers, have received instructions to purchase 10,000 more good animals. A prominent mule dealer of Austin, Texas, recently received a telegram from one of the representatives, now in New Orleans, offering good prices for as many animals as could be furnished him. The shipment of 2000 mules from Fannin County to South Africa by way of New Orleans began December 14th.

Tuberculosis among Belgian Cattle. Consul Roosevelt, of Brussels, August 22d (Consular Reports, No. 230) transmits the following :

The Minister of Agriculture has invited provincial agricultural societies to make known to farmers and cattle breeders through the medium of lectures, notices, and circulars the means of fighting the increase of tuberculosis among cattle. Their attention is to be notably drawn to the hygiene of stables, as in many districts, especially the Campine and Ardennes, the stables are very badly kept. The peasants are afraid of leaving the doors of the stable open on account of draughts, which they think highly injurious to he cattle, and it is difficult to make them understand that stables are contaminated when deprived of fresh air. Also very little attention is given to cleanliness. Frequently the litter is allowed to accumulate for weeks and even months until the unfortunate animals stand knee-deep in it. Under such conditions it is easy to understand that such stables become propagating beds for the germs of tuberculosis. As it is difficult to make the peasants understand that upon the cleanliness of the stable largely depends the eradication of the disease among their cattle, it has been determined to organize competition, awarding premiums to farmers taking good care of their cattle and stables.—*Sanitarian*, January, 1900.

The Secretary of Agriculture in his report just issued, referring to the work of the Bureau of Animal Industry, says :

The work of this Bureau increases rapidly. Meat inspection was conducted last year at one hundred and thirty-eight abattoirs in forty-one cities. The ante-mortem inspections were 53,223,176, while the number in 1892 was 3,809,459. The third year of experimentation with hog-cholera shows that from 75 to 80 per cent. of hogs injected with serum are saved. Encouraging results have come from the introduction of dairy products into foreign markets. The Department sends shipments abroad for the purpose of ascertaining the facts regarding such products, these facts are published and commerce naturally follows.

The Bureau's work is summed up in a number of forceful recommendations, of great importance from a public health point of view, and of vast moment to the commercial industries involved.

It is a source of much pleasure and surprise to note the many directions of work covered by the Department of Agriculture, and how far-reaching must be the results obtained in solving the many problems wrapped up in nature, which are ofttimes barriers to the welfare and advancement of a large body of our people.

A better knowledge of this great work done by this department in its economic relations with our people would add much to its better support and make of force the many recommendations made by the Secretary.

Rochester, N. Y., has repealed a recent city ordinance relative to a milk standard based upon the number of bacteria per cubic centimetre of milk. It is said to have conflicted with laws of the Department of Agriculture of the State.

